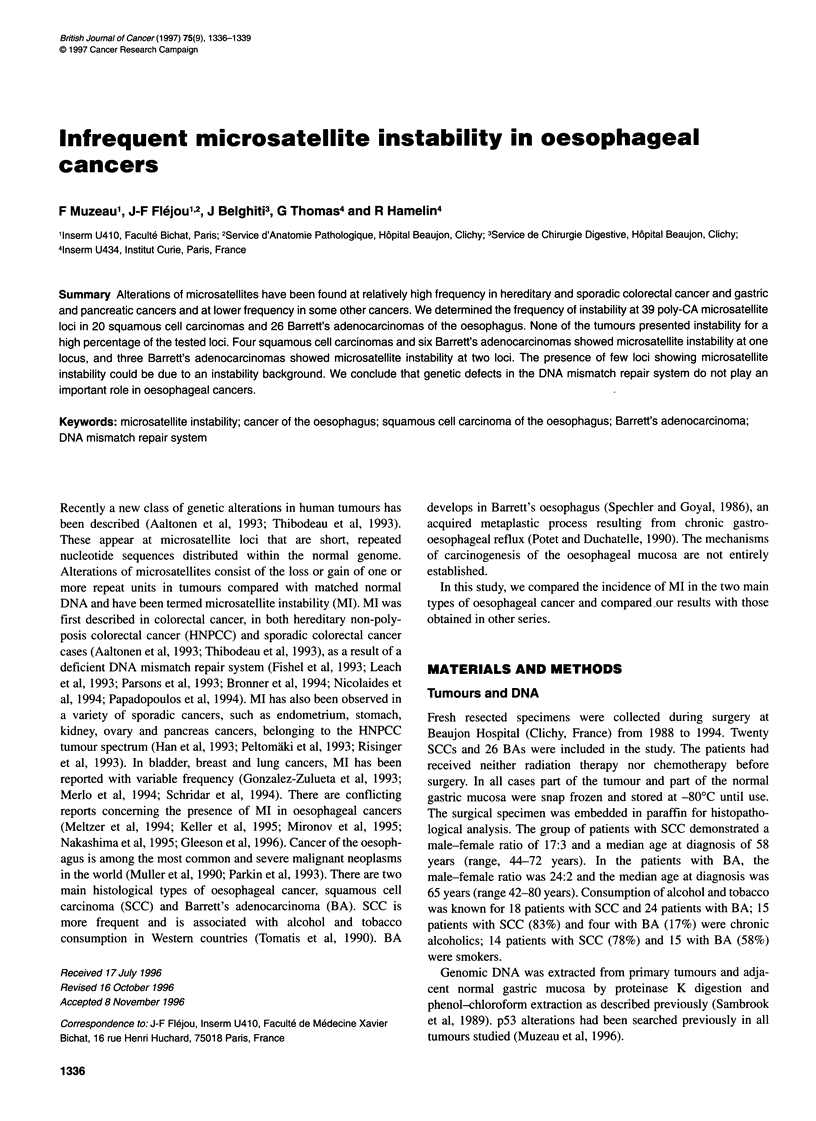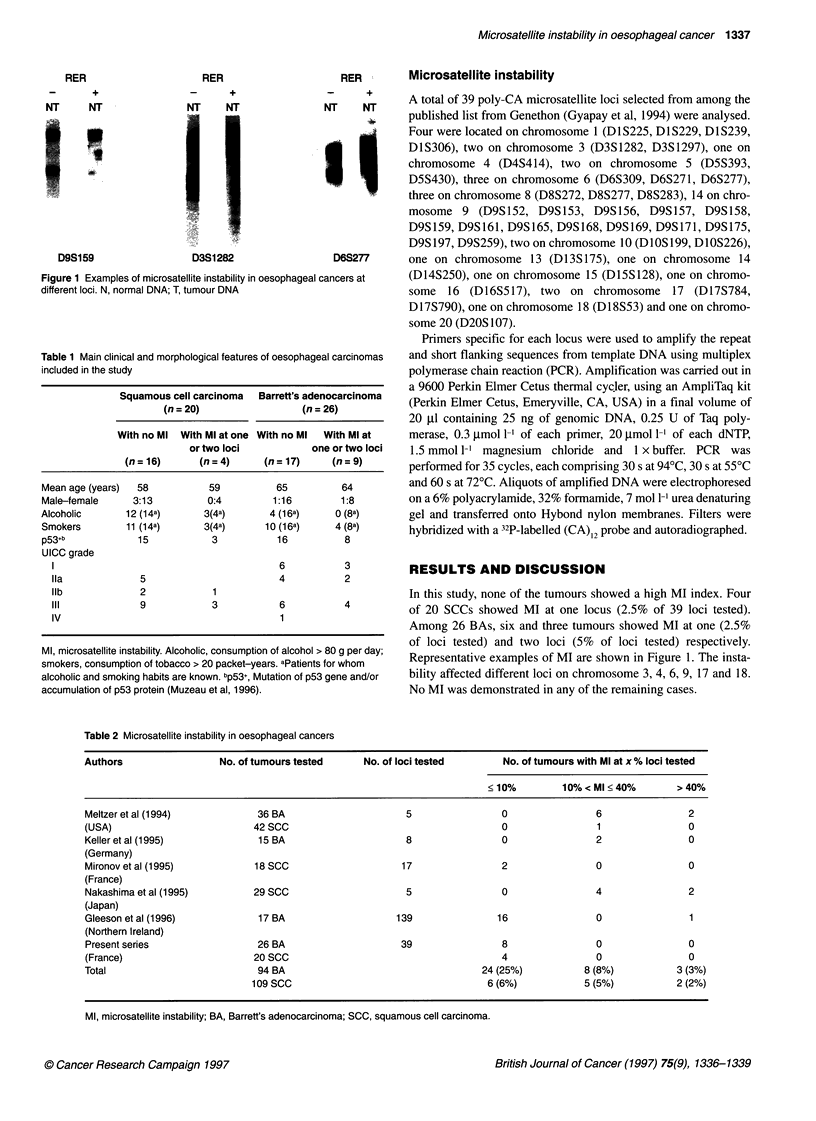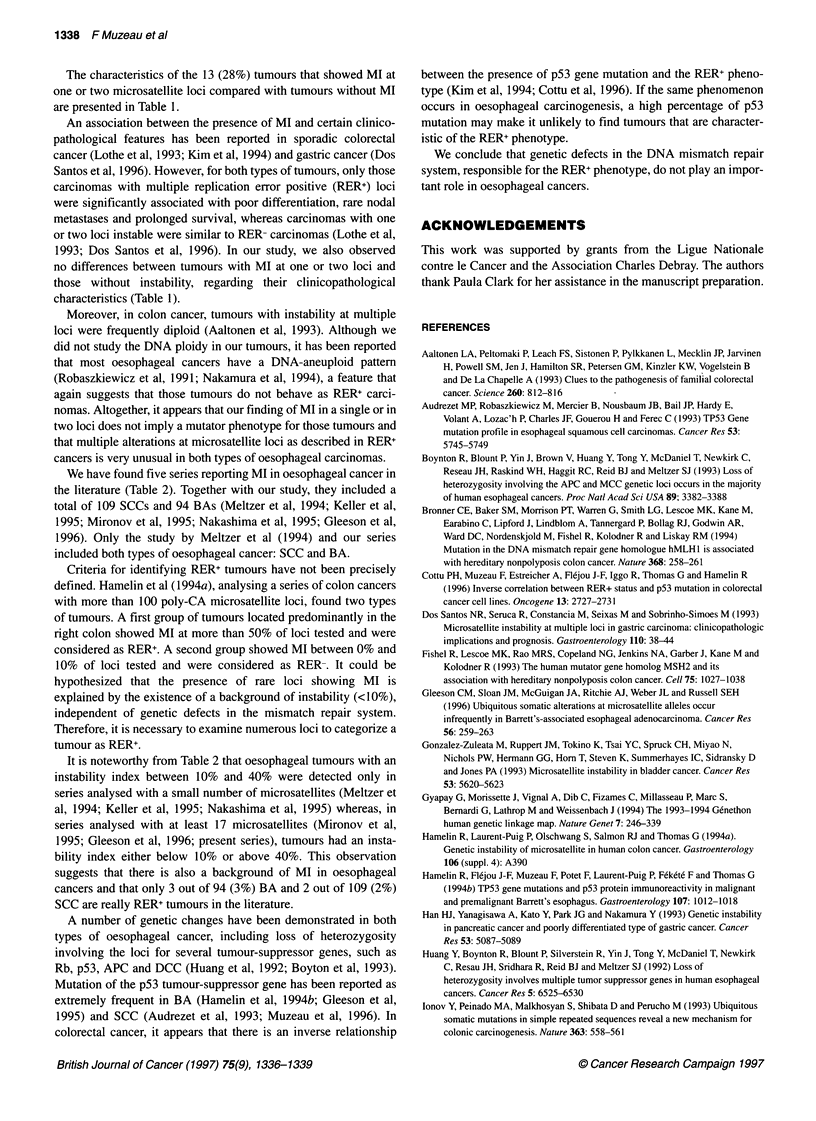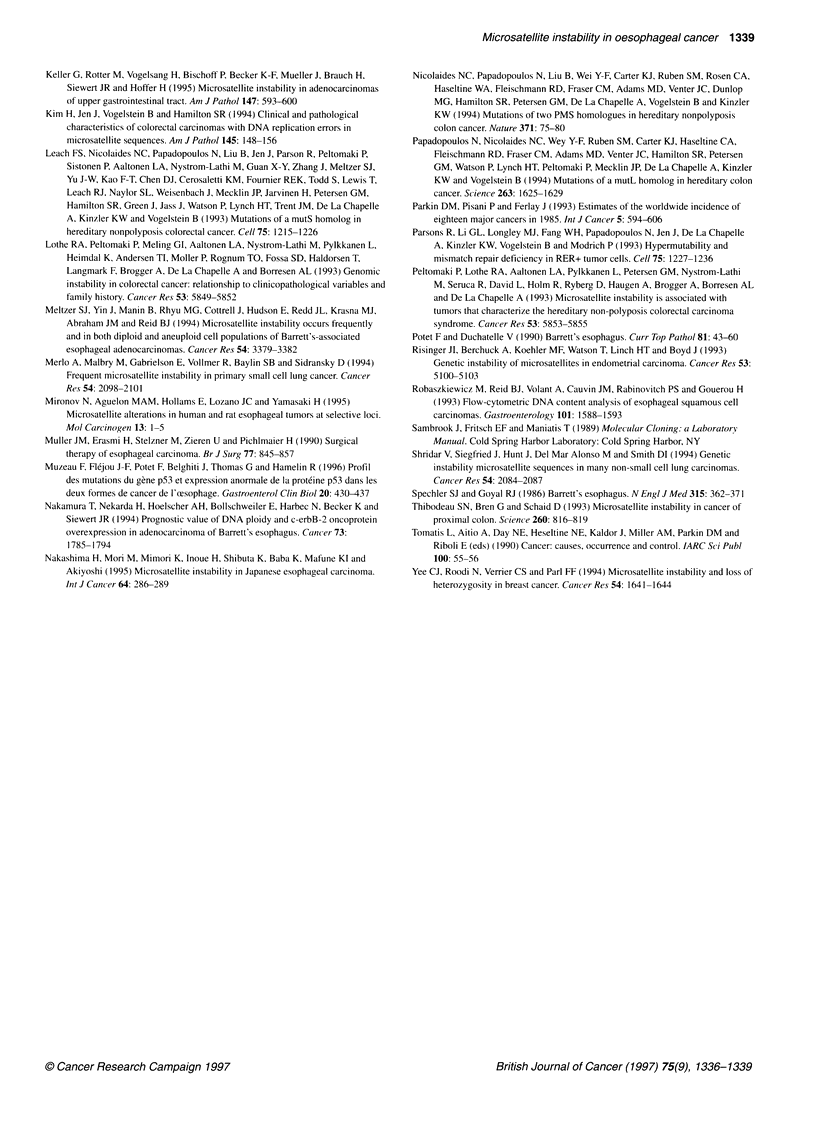# Infrequent microsatellite instability in oesophageal cancers.

**DOI:** 10.1038/bjc.1997.226

**Published:** 1997

**Authors:** F. Muzeau, J. F. FlÃ©jou, J. Belghiti, G. Thomas, R. Hamelin

**Affiliations:** Inserm U410, FacultÃ© Bichat, Paris, France.

## Abstract

**Images:**


British Journal of Cancer (1997) 75(9), 1336–1339
© 1997 Cancer Research Campaign

### Infrequent microsatellite instability in oesophageal cancers

**F Muzeau[1], J-F Fléjou[1,2], J Belghiti[3], G Thomas[4] and R Hamelin[4]**

[1]Inserm U410, Faculté Bichat, Paris; [2]Service d'Anatomie Pathologique, Hôpital Beaujon, Clichy; [3]Service de Chirurgie Digestive, Hôpital Beaujon, Clichy; [4]Inserm U434, Institut Curie, Paris, France

**Summary** Alterations of microsatellites have been found at relatively high frequency in hereditary and sporadic colorectal cancer and gastric and pancreatic cancers and at lower frequency in some other cancers. We determined the frequency of instability at 39 poly-CA microsatellite loci in 20 squamous cell carcinomas and 26 Barrett's adenocarcinomas of the oesophagus. None of the tumours presented instability for a high percentage of the tested loci. Four squamous cell carcinomas and six Barrett's adenocarcinomas showed microsatellite instability at one locus, and three Barrett's adenocarcinomas showed microsatellite instability at two loci. The presence of few loci showing microsatellite instability could be due to an instability background. We conclude that genetic defects in the DNA mismatch repair system do not play an important role in oesophageal cancers.

**Keywords:** microsatellite instability; cancer of the oesophagus; squamous cell carcinoma of the oesophagus; Barrett's adenocarcinoma; DNA mismatch repair system

Recently a new class of genetic alterations in human tumours has been described (Aaltonen et al, 1993; Thibodeau et al, 1993). These appear at microsatellite loci that are short, repeated nucleotide sequences distributed within the normal genome. Alterations of microsatellites consist of the loss or gain of one or more repeat units in tumours compared with matched normal DNA and have been termed microsatellite instability (MI). MI was first described in colorectal cancer, in both hereditary non-polyposis colorectal cancer (HNPCC) and sporadic colorectal cancer cases (Aaltonen et al, 1993; Thibodeau et al, 1993), as a result of a deficient DNA mismatch repair system (Fishel et al, 1993; Leach et al, 1993; Parsons et al, 1993; Bronner et al, 1994; Nicolaides et al, 1994; Papadopoulos et al, 1994). MI has also been observed in a variety of sporadic cancers, such as endometrium, stomach, kidney, ovary and pancreas cancers, belonging to the HNPCC tumour spectrum (Han et al, 1993; Peltomäki et al, 1993; Risinger et al, 1993). In bladder, breast and lung cancers, MI has been reported with variable frequency (Gonzalez-Zulueta et al, 1993; Merlo et al, 1994; Schridar et al, 1994). There are conflicting reports concerning the presence of MI in oesophageal cancers (Meltzer et al, 1994; Keller et al, 1995; Mironov et al, 1995; Nakashima et al, 1995; Gleeson et al, 1996). Cancer of the oesophagus is among the most common and severe malignant neoplasms in the world (Muller et al, 1990; Parkin et al, 1993). There are two main histological types of oesophageal cancer, squamous cell carcinoma (SCC) and Barrett's adenocarcinoma (BA). SCC is more frequent and is associated with alcohol and tobacco consumption in Western countries (Tomatis et al, 1990). BA develops in Barrett's oesophagus (Spechler and Goyal, 1986), an acquired metaplastic process resulting from chronic gastro-oesophageal reflux (Potet and Duchatelle, 1990). The mechanisms of carcinogenesis of the oesophageal mucosa are not entirely established.

In this study, we compared the incidence of MI in the two main types of oesophageal cancer and compared our results with those obtained in other series.

*Received 17 July 1996*
*Revised 16 October 1996*
*Accepted 8 November 1996*

*Correspondence to:* J-F Fléjou, Inserm U410, Faculté de Médecine Xavier Bichat, 16 rue Henri Huchard, 75018 Paris, France

### MATERIALS AND METHODS

### Tumours and DNA

Fresh resected specimens were collected during surgery at Beaujon Hospital (Clichy, France) from 1988 to 1994. Twenty SCCs and 26 BAs were included in the study. The patients had received neither radiation therapy nor chemotherapy before surgery. In all cases part of the tumour and part of the normal gastric mucosa were snap frozen and stored at –80°C until use. The surgical specimen was embedded in paraffin for histopathological analysis. The group of patients with SCC demonstrated a male–female ratio of 17:3 and a median age at diagnosis of 58 years (range, 44–72 years). In the patients with BA, the male–female ratio was 24:2 and the median age at diagnosis was 65 years (range 42–80 years). Consumption of alcohol and tobacco was known for 18 patients with SCC and 24 patients with BA; 15 patients with SCC (83%) and four with BA (17%) were chronic alcoholics; 14 patients with SCC (78%) and 15 with BA (58%) were smokers.

Genomic DNA was extracted from primary tumours and adjacent normal gastric mucosa by proteinase K digestion and phenol–chloroform extraction as described previously (Sambrook et al, 1989). p53 alterations had been searched previously in all tumours studied (Muzeau et al, 1996).

**Figure 1** Examples of microsatellite instability in oesophageal cancers at different loci. N, normal DNA; T, tumour DNA

**Table 1** Main clinical and morphological features of oesophageal carcinomas included in the study

| | Squamous cell carcinoma ($n$ = 20) | | Barrett's adenocarcinoma ($n$ = 26) | |
|---|---|---|---|---|
| | With no MI ($n$ = 16) | With MI at one or two loci ($n$ = 4) | With no MI ($n$ = 17) | With MI at one or two loci ($n$ = 9) |
| Mean age (years) | 58 | 59 | 65 | 64 |
| Male–female | 3:13 | 0:4 | 1:16 | 1:8 |
| Alcoholic | 12 (14[a]) | 3(4[a]) | 4 (16[a]) | 0 (8[a]) |
| Smokers | 11 (14[a]) | 3(4[a]) | 10 (16[a]) | 4 (8[a]) |
| p53+[b] | 15 | 3 | 16 | 8 |
| UICC grade | | | | |
| I | | | 6 | 3 |
| IIa | 5 | | 4 | 2 |
| IIb | 2 | 1 | | |
| III | 9 | 3 | 6 | 4 |
| IV | | | 1 | |

MI, microsatellite instability. Alcoholic, consumption of alcohol > 80 g per day; smokers, consumption of tobacco > 20 packet–years. [a]Patients for whom alcoholic and smoking habits are known. [b]p53+, Mutation of p53 gene and/or accumulation of p53 protein (Muzeau et al, 1996).

### Microsatellite instability

A total of 39 poly-CA microsatellite loci selected from among the published list from Genethon (Gyapay et al, 1994) were analysed. Four were located on chromosome 1 (D1S225, D1S229, D1S239, D1S306), two on chromosome 3 (D3S1282, D3S1297), one on chromosome 4 (D4S414), two on chromosome 5 (D5S393, D5S430), three on chromosome 6 (D6S309, D6S271, D6S277), three on chromosome 8 (D8S272, D8S277, D8S283), 14 on chromosome 9 (D9S152, D9S153, D9S156, D9S157, D9S158, D9S159, D9S161, D9S165, D9S168, D9S169, D9S171, D9S175, D9S197, D9S259), two on chromosome 10 (D10S199, D10S226), one on chromosome 13 (D13S175), one on chromosome 14 (D14S250), one on chromosome 15 (D15S128), one on chromosome 16 (D16S517), two on chromosome 17 (D17S784, D17S790), one on chromosome 18 (D18S53) and one on chromosome 20 (D20S107).

Primers specific for each locus were used to amplify the repeat and short flanking sequences from template DNA using multiplex polymerase chain reaction (PCR). Amplification was carried out in a 9600 Perkin Elmer Cetus thermal cycler, using an AmpliTaq kit (Perkin Elmer Cetus, Emeryville, CA, USA) in a final volume of 20 μl containing 25 ng of genomic DNA, 0.25 U of Taq polymerase, 0.3 $\mu mol\ l^{-1}$ of each primer, 20 $\mu mol\ l^{-1}$ of each dNTP, 1.5 $mmol\ l^{-1}$ magnesium chloride and $1 \times$ buffer. PCR was performed for 35 cycles, each comprising 30 s at 94°C, 30 s at 55°C and 60 s at 72°C. Aliquots of amplified DNA were electrophoresed on a 6% polyacrylamide, 32% formamide, 7 $mol\ l^{-1}$ urea denaturing gel and transferred onto Hybond nylon membranes. Filters were hybridized with a $^{32}P$-labelled $(CA)_{12}$ probe and autoradiographed.

### RESULTS AND DISCUSSION

In this study, none of the tumours showed a high MI index. Four of 20 SCCs showed MI at one locus (2.5% of 39 loci tested). Among 26 BAs, six and three tumours showed MI at one (2.5% of loci tested) and two loci (5% of loci tested) respectively. Representative examples of MI are shown in Figure 1. The instability affected different loci on chromosome 3, 4, 6, 9, 17 and 18. No MI was demonstrated in any of the remaining cases.

**Table 2** Microsatellite instability in oesophageal cancers

| Authors | No. of tumours tested | No. of loci tested | No. of tumours with MI at $x$ % loci tested | | |
|---|---|---|---|---|---|
| | | | ≤ 10% | 10% < MI ≤ 40% | > 40% |
| Meltzer et al (1994) | 36 BA | 5 | 0 | 6 | 2 |
| (USA) | 42 SCC | | 0 | 1 | 0 |
| Keller et al (1995) (Germany) | 15 BA | 8 | 0 | 2 | 0 |
| Mironov et al (1995) (France) | 18 SCC | 17 | 2 | 0 | 0 |
| Nakashima et al (1995) (Japan) | 29 SCC | 5 | 0 | 4 | 2 |
| Gleeson et al (1996) (Northern Ireland) | 17 BA | 139 | 16 | 0 | 1 |
| Present series | 26 BA | 39 | 8 | 0 | 0 |
| (France) | 20 SCC | | 4 | 0 | 0 |
| Total | 94 BA | | 24 (25%) | 8 (8%) | 3 (3%) |
| | 109 SCC | | 6 (6%) | 5 (5%) | 2 (2%) |

MI, microsatellite instability; BA, Barrett's adenocarcinoma; SCC, squamous cell carcinoma.

© Cancer Research Campaign 1997

The characteristics of the 13 (28%) tumours that showed MI at one or two microsatellite loci compared with tumours without MI are presented in Table 1.

An association between the presence of MI and certain clinicopathological features has been reported in sporadic colorectal cancer (Lothe et al, 1993; Kim et al, 1994) and gastric cancer (Dos Santos et al, 1996). However, for both types of tumours, only those carcinomas with multiple replication error positive ($RER^+$) loci were significantly associated with poor differentiation, rare nodal metastases and prolonged survival, whereas carcinomas with one or two loci instable were similar to $RER^-$ carcinomas (Lothe et al, 1993; Dos Santos et al, 1996). In our study, we also observed no differences between tumours with MI at one or two loci and those without instability, regarding their clinicopathological characteristics (Table 1).

Moreover, in colon cancer, tumours with instability at multiple loci were frequently diploid (Aaltonen et al, 1993). Although we did not study the DNA ploidy in our tumours, it has been reported that most oesophageal cancers have a DNA-aneuploid pattern (Robaszkiewicz et al, 1991; Nakamura et al, 1994), a feature that again suggests that those tumours do not behave as $RER^+$ carcinomas. Altogether, it appears that our finding of MI in a single or in two loci does not imply a mutator phenotype for those tumours and that multiple alterations at microsatellite loci as described in $RER^+$ cancers is very unusual in both types of oesophageal carcinomas.

We have found five series reporting MI in oesophageal cancer in the literature (Table 2). Together with our study, they included a total of 109 SCCs and 94 BAs (Meltzer et al, 1994; Keller et al, 1995; Mironov et al, 1995; Nakashima et al, 1995; Gleeson et al, 1996). Only the study by Meltzer et al (1994) and our series included both types of oesophageal cancer: SCC and BA.

Criteria for identifying $RER^+$ tumours have not been precisely defined. Hamelin et al (1994*a*), analysing a series of colon cancers with more than 100 poly-CA microsatellite loci, found two types of tumours. A first group of tumours located predominantly in the right colon showed MI at more than 50% of loci tested and were considered as $RER^+$. A second group showed MI between 0% and 10% of loci tested and were considered as $RER^-$. It could be hypothesized that the presence of rare loci showing MI is explained by the existence of a background of instability (<10%), independent of genetic defects in the mismatch repair system. Therefore, it is necessary to examine numerous loci to categorize a tumour as $RER^+$.

It is noteworthy from Table 2 that oesophageal tumours with an instability index between 10% and 40% were detected only in series analysed with a small number of microsatellites (Meltzer et al, 1994; Keller et al, 1995; Nakashima et al, 1995) whereas, in series analysed with at least 17 microsatellites (Mironov et al, 1995; Gleeson et al, 1996; present series), tumours had an instability index either below 10% or above 40%. This observation suggests that there is also a background of MI in oesophageal cancers and that only 3 out of 94 (3%) BA and 2 out of 109 (2%) SCC are really $RER^+$ tumours in the literature.

A number of genetic changes have been demonstrated in both types of oesophageal cancer, including loss of heterozygosity involving the loci for several tumour-suppressor genes, such as Rb, p53, APC and DCC (Huang et al, 1992; Boyton et al, 1993). Mutation of the p53 tumour-suppressor gene has been reported as extremely frequent in BA (Hamelin et al, 1994*b*; Gleeson et al, 1995) and SCC (Audrezet et al, 1993; Muzeau et al, 1996). In colorectal cancer, it appears that there is an inverse relationship between the presence of p53 gene mutation and the $RER^+$ phenotype (Kim et al, 1994; Cottu et al, 1996). If the same phenomenon occurs in oesophageal carcinogenesis, a high percentage of p53 mutation may make it unlikely to find tumours that are characteristic of the $RER^+$ phenotype.

We conclude that genetic defects in the DNA mismatch repair system, responsible for the $RER^+$ phenotype, do not play an important role in oesophageal cancers.

### ACKNOWLEDGEMENTS

This work was supported by grants from the Ligue Nationale contre le Cancer and the Association Charles Debray. The authors thank Paula Clark for her assistance in the manuscript preparation.

### REFERENCES

Aaltonen LA, Peltomaki P, Leach FS, Sistonen P, Pylkkanen L, Mecklin JP, Jarvinen H, Powell SM, Jen J, Hamilton SR, Petersen GM, Kinzler KW, Vogelstein B and De La Chapelle A (1993) Clues to the pathogenesis of familial colorectal cancer. *Science* **260**: 812–816

Audrezet MP, Robaszkiewicz M, Mercier B, Nousbaum JB, Bail JP, Hardy E, Volant A, Lozac'h P, Charles JF, Gouerou H and Ferec C (1993) TP53 Gene mutation profile in esophageal squamous cell carcinomas. *Cancer Res* **53**: 5745–5749

Boynton R, Blount P, Yin J, Brown V, Huang Y, Tong Y, McDaniel T, Newkirk C, Reseau JH, Raskind WH, Haggit RC, Reid BJ and Meltzer SJ (1993) Loss of heterozygosity involving the APC and MCC genetic loci occurs in the majority of human esophageal cancers. *Proc Natl Acad Sci USA* **89**; 3382–3388

Bronner CE, Baker SM, Morrison PT, Warren G, Smith LG, Lescoe MK, Kane M, Earabino C, Lipford J, Lindblom A, Tannergard P, Bollag RJ, Godwin AR, Ward DC, Nordenskjold M, Fishel R, Kolodner R and Liskay RM (1994) Mutation in the DNA mismatch repair gene homologue hMLH1 is associated with hereditary nonpolyposis colon cancer. *Nature* **368**: 258–261

Cottu PH, Muzeau F, Estreicher A, Fléjou J-F, Iggo R, Thomas G and Hamelin R (1996) Inverse correlation between RER+ status and p53 mutation in colorectal cancer cell lines. *Oncogene* **13**: 2727–2731

Dos Santos NR, Seruca R, Constancia M, Seixas M and Sobrinho-Simoes M (1993) Microsatellite instability at multiple loci in gastric carcinoma: clinicopathologic implications and prognosis. *Gastroenterology* **110**: 38–44

Fishel R, Lescoe MK, Rao MRS, Copeland NG, Jenkins NA, Garber J, Kane M and Kolodner R (1993) The human mutator gene homolog MSH2 and its association with hereditary nonpolyposis colon cancer. *Cell* **75**: 1027–1038

Gleeson CM, Sloan JM, McGuigan JA, Ritchie AJ, Weber JL and Russell SEH (1996) Ubiquitous somatic alterations at microsatellite alleles occur infrequently in Barrett's-associated esophageal adenocarcinoma. *Cancer Res* **56**: 259–263

Gonzalez-Zuleata M, Ruppert JM, Tokino K, Tsai YC, Spruck CH, Miyao N, Nichols PW, Hermann GG, Horn T, Steven K, Summerhayes IC, Sidransky D and Jones PA (1993) Microsatellite instability in bladder cancer. *Cancer Res* **53**: 5620–5623

Gyapay G, Morissette J, Vignal A, Dib C, Fizames C, Millasseau P, Marc S, Bernardi G, Lathrop M and Weissenbach J (1994) The 1993–1994 Génethon human genetic linkage map. *Nature Genet* **7**: 246–339

Hamelin R, Laurent-Puig P, Olschwang S, Salmon RJ and Thomas G (1994*a*). Genetic instability of microsatellite in human colon cancer. *Gastroenterology* **106** (suppl. 4): A390

Hamelin R, Fléjou J-F, Muzeau F, Potet F, Laurent-Puig P, Fékété F and Thomas G (1994*b*) TP53 gene mutations and p53 protein immunoreactivity in malignant and premalignant Barrett's esophagus. *Gastroenterology* **107**: 1012–1018

Han HJ, Yanagisawa A, Kato Y, Park JG and Nakamura Y (1993) Genetic instability in pancreatic cancer and poorly differentiated type of gastric cancer. *Cancer Res* **53**: 5087–5089

Huang Y, Boynton R, Blount P, Silverstein R, Yin J, Tong Y, McDaniel T, Newkirk C, Resau JH, Sridhara R, Reid BJ and Meltzer SJ (1992) Loss of heterozygosity involves multiple tumor suppressor genes in human esophageal cancers. *Cancer Res* **5**: 6525–6530

Ionov Y, Peinado MA, Malkhosyan S, Shibata D and Perucho M (1993) Ubiquitous somatic mutations in simple repeated sequences reveal a new mechanism for colonic carcinogenesis. *Nature* **363**: 558–561

© Cancer Research Campaign 1997

Keller G, Rotter M, Vogelsang H, Bischoff P, Becker K-F, Mueller J, Brauch H, Siewert JR and Hoffer H (1995) Microsatellite instability in adenocarcinomas of upper gastrointestinal tract. *Am J Pathol* **147**: 593–600

Kim H, Jen J, Vogelstein B and Hamilton SR (1994) Clinical and pathological characteristics of colorectal carcinomas with DNA replication errors in microsatellite sequences. *Am J Pathol* **145**: 148–156

Leach FS, Nicolaides NC, Papadopoulos N, Liu B, Jen J, Parson R, Peltomaki P, Sistonen P, Aaltonen LA, Nystrom-Lathi M, Guan X-Y, Zhang J, Meltzer SJ, Yu J-W, Kao F-T, Chen DJ, Cerosaletti KM, Fournier REK, Todd S, Lewis T, Leach RJ, Naylor SL, Weisenbach J, Mecklin JP, Jarvinen H, Petersen GM, Hamilton SR, Green J, Jass J, Watson P, Lynch HT, Trent JM, De La Chapelle A, Kinzler KW and Vogelstein B (1993) Mutations of a mutS homolog in hereditary nonpolyposis colorectal cancer. *Cell* **75**: 1215–1226

Lothe RA, Peltomaki P, Meling GI, Aaltonen LA, Nystrom-Lathi M, Pylkkanen L, Heimdal K, Andersen TI, Moller P, Rognum TO, Fossa SD, Haldorsen T, Langmark F, Brogger A, De La Chapelle A and Borresen AL (1993) Genomic instability in colorectal cancer: relationship to clinicopathological variables and family history. *Cancer Res* **53**: 5849–5852

Meltzer SJ, Yin J, Manin B, Rhyu MG, Cottrell J, Hudson E, Redd JL, Krasna MJ, Abraham JM and Reid BJ (1994) Microsatellite instability occurs frequently and in both diploid and aneuploid cell populations of Barrett's-associated esophageal adenocarcinomas. *Cancer Res* **54**: 3379–3382

Merlo A, Malbry M, Gabrielson E, Vollmer R, Baylin SB and Sidransky D (1994) Frequent microsatellite instability in primary small cell lung cancer. *Cancer Res* **54**: 2098–2101

Mironov N, Aguelon MAM, Hollams E, Lozano JC and Yamasaki H (1995) Microsatellite alterations in human and rat esophageal tumors at selective loci. *Mol Carcinogen* **13**: 1–5

Muller JM, Erasmi H, Stelzner M, Zieren U and Pichlmaier H (1990) Surgical therapy of esophageal carcinoma. *Br J Surg* **77**: 845–857

Muzeau F, Fléjou J-F, Potet F, Belghiti J, Thomas G and Hamelin R (1996) Profil des mutations du gène p53 et expression anormale de la protéine p53 dans les deux formes de cancer de l'œsophage. *Gastroenterol Clin Biol* **20**: 430–437

Nakamura T, Nekarda H, Hoelscher AH, Bollschweiler E, Harbec N, Becker K and Siewert JR (1994) Prognostic value of DNA ploidy and c-erbB-2 oncoprotein overexpression in adenocarcinoma of Barrett's esophagus. *Cancer* **73**: 1785–1794

Nakashima H, Mori M, Mimori K, Inoue H, Shibuta K, Baba K, Mafune KI and Akiyoshi (1995) Microsatellite instability in Japanese esophageal carcinoma. *Int J Cancer* **64**: 286–289

Nicolaides NC, Papadopoulos N, Liu B, Wei Y-F, Carter KJ, Ruben SM, Rosen CA, Haseltine WA, Fleischmann RD, Fraser CM, Adams MD, Venter JC, Dunlop MG, Hamilton SR, Petersen GM, De La Chapelle A, Vogelstein B and Kinzler KW (1994) Mutations of two PMS homologues in hereditary nonpolyposis colon cancer. *Nature* **371**: 75–80

Papadopoulos N, Nicolaides NC, Wey Y-F, Ruben SM, Carter KJ, Haseltine CA, Fleischmann RD, Fraser CM, Adams MD, Venter JC, Hamilton SR, Petersen GM, Watson P, Lynch HT, Peltomaki P, Mecklin JP, De La Chapelle A, Kinzler KW and Vogelstein B (1994) Mutations of a mutL homolog in hereditary colon cancer. *Science* **263**: 1625–1629

Parkin DM, Pisani P and Ferlay J (1993) Estimates of the worldwide incidence of eighteen major cancers in 1985. *Int J Cancer* **5**: 594–606

Parsons R, Li GL, Longley MJ, Fang WH, Papadopoulos N, Jen J, De La Chapelle A, Kinzler KW, Vogelstein B and Modrich P (1993) Hypermutability and mismatch repair deficiency in RER+ tumor cells. *Cell* **75**: 1227–1236

Peltomaki P, Lothe RA, Aaltonen LA, Pylkkanen L, Petersen GM, Nystrom-Lathi M, Seruca R, David L, Holm R, Ryberg D, Haugen A, Brogger A, Borresen AL and De La Chapelle A (1993) Microsatellite instability is associated with tumors that characterize the hereditary non-polyposis colorectal carcinoma syndrome. *Cancer Res* **53**: 5853–5855

Potet F and Duchatelle V (1990) Barrett's esophagus. *Curr Top Pathol* **81**: 43–60

Risinger JI, Berchuck A, Koehler MF, Watson T, Linch HT and Boyd J (1993) Genetic instability of microsatellites in endometrial carcinoma. *Cancer Res* **53**: 5100–5103

Robaszkiewicz M, Reid BJ, Volant A, Cauvin JM, Rabinovitch PS and Gouerou H (1993) Flow-cytometric DNA content analysis of esophageal squamous cell carcinomas. *Gastroenterology* **101**: 1588–1593

Sambrook J, Fritsch EF and Maniatis T (1989) *Molecular Cloning: a Laboratory Manual*. Cold Spring Harbor Laboratory: Cold Spring Harbor, NY

Shridar V, Siegfried J, Hunt J, Del Mar Alonso M and Smith DI (1994) Genetic instability microsatellite sequences in many non-small cell lung carcinomas. *Cancer Res* **54**: 2084–2087

Spechler SJ and Goyal RJ (1986) Barrett's esophagus. *N Engl J Med* **315**: 362–371

Thibodeau SN, Bren G and Schaid D (1993) Microsatellite instability in cancer of proximal colon. *Science* **260**: 816–819

Tomatis L, Aitio A, Day NE, Heseltine NE, Kaldor J, Miller AM, Parkin DM and Riboli E (eds) (1990) Cancer: causes, occurrence and control. *IARC Sci Publ* **100**: 55–56

Yee CJ, Roodi N, Verrier CS and Parl FF (1994) Microsatellite instability and loss of heterozygosity in breast cancer. *Cancer Res* **54**: 1641–1644

© Cancer Research Campaign 1997